# Prevalence and Risk Factors for Multidrug-Resistant Organisms Colonization in Long-Term Care Facilities Around the World: A Review

**DOI:** 10.3390/antibiotics10060680

**Published:** 2021-06-07

**Authors:** Ángel Rodríguez-Villodres, Cecilia Martín-Gandul, Germán Peñalva, Ana Belén Guisado-Gil, Juan Carlos Crespo-Rivas, María Eugenia Pachón-Ibáñez, José Antonio Lepe, José Miguel Cisneros

**Affiliations:** 1 Clinical Unit of Infectious Diseases, Microbiology and Preventive Medicine, Institute of Biomedicine of Seville (IBiS), University of Seville/CSIC/University Hospital Virgen del Rocío, 41013 Seville, Spain; anrovi1797@gmail.com (Á.R.-V.); cecilia3778@hotmail.com (C.M.-G.); german.penalva@gmail.com (G.P.); anaguigil@gmail.com (A.B.G.-G.); jccresporivas@gmail.com (J.C.C.-R.); mpachon-ibis@us.es (M.E.P.-I.); josealepe@gmail.com (J.A.L.); 2 Department of Pharmacy, University Hospital Virgen del Rocío, 41013 Seville, Spain

**Keywords:** multidrug-resistant organism, long term care facilities, nursing homes, prevalence, risk factors, antimicrobial stewardship

## Abstract

Elderly people confined to chronic care facilities face an increased risk of acquiring infections by multidrug-resistant organisms (MDROs). This review presents the current knowledge of the prevalence and risk factors for colonization by MDROs in long-term care facilities (LTCF), thereby providing a useful reference to establish objectives for implementing successful antimicrobial stewardship programs (ASPs). We searched in PubMed and Scopus for studies examining the prevalence of MDROs and/or risk factors for the acquisition of MDROs in LTCF. One hundred and thirty-four studies published from 1987 to 2020 were included. The prevalence of MDROs in LTCF varies between the different continents, where Asia reported the highest prevalence of extended-spectrum ß-lactamase (ESBL) *Enterobacterales* (71.6%), carbapenem resistant (CR) *Enterobacterales* (6.9%) and methicillin-resistant *Staphylococcus aureus* (MRSA) (25.6%) and North America the highest prevalence to MDR *Pseudomonas aeruginosa* (5.4%), MDR *Acinetobacter baumannii* (15.0%), vancomycin-resistant *Enterococcus* spp. (VRE) (4.0%), and *Clostridioides difficile* (26.1%). Furthermore, MDRO prevalence has experienced changes over time, with increases in MDR *P. aeruginosa* and extended spectrum ß-lactamase producing *Enterobacterales* observed starting in 2015 and decreases of CR *Enterobacterales*, MDR *A. baumannii*, VRE, MRSA and *C. difficile.* Several risk factors have been found, such as male sex, chronic wounds, the use of medical devices, and previous antibiotic use. The last of these aspects represents one of the most important modifiable factors for reducing colonization with MDROs through implementing ASPs in LTCF.

## 1. Introduction

Older adults confined to chronic care facilities are known to have an increased risk of acquiring infections, most commonly those of the skin, soft tissues, respiratory and urinary tracts, as well as gastroenteritis [1]. The recent COVID-19 outbreak has underlined the vulnerability of this group around the world [2], and the condition of frailty is gaining international attention. According to the Organization for Economic Co-operation and Development (OECD), it is expected that the population aged from 65 to 79 years will rise from about 10% in 2010 to about 15% in 2050 (https://www.oecd.org/els/health-systems/47884543.pdf; accessed on 14 December 2020). Thus, current trends in the world’s demographic structure indicate increasing requirements in long-term care settings.

The emergence of multidrug-resistant organisms (MDROs) is a major public health concern [3]. Bacteria such as extended-spectrum ß-lactamase (ESBL) *Escherichia coli*, ESBL *Klebsiella pneumoniae*, carbapenem-resistant (CR) *Enterobacterales*, methicillin-resistant *Staphylococcus aureus* (MRSA)**, vancomycin-resistant *Enterococcus* spp. (VRE)**, and multidrug-resistant (MDR) non-fermenting species such as *Pseudomonas aeruginosa* and *Acinetobacter baumannii* are increasing in prevalence [4,5,6,7]. Although it is not an MDRO, *Clostridioides difficile* represents a worldwide public health concern, as it is one of the major causes of antibiotic-associated infections in healthcare settings, especially among older people living in nursing homes (NH) [8,9].

Polypharmacy and inappropriate prescriptions are well-known risk factors for adverse drug reactions, which commonly cause poor clinical outcomes in older people. Antimicrobial resistance is a major negative event resulting from inappropriate prescriptions of antimicrobials [10].

Antimicrobial stewardship programs (ASPs) have been widely implemented in hospitals, with considerable evidence of their impacts on prescription rates and some evidence that they reduce antibiotic-resistant infections [11]. Elderly care facilities and NH could also benefit from these programs. Knowledge of the epidemiology of MDROs at a local and global level is key to implementing successful antimicrobial stewardship intervention. The purpose of this narrative review is to present current knowledge on the prevalence and risk factors for colonization by MDROs in long-term care facilities (LTCF), along with epidemiological data to facilitate the establishment of objectives for implementing successful ASPs in this setting.

## 2. Results and Discussion

### 2.1. Studies Included

**Figure 1:**
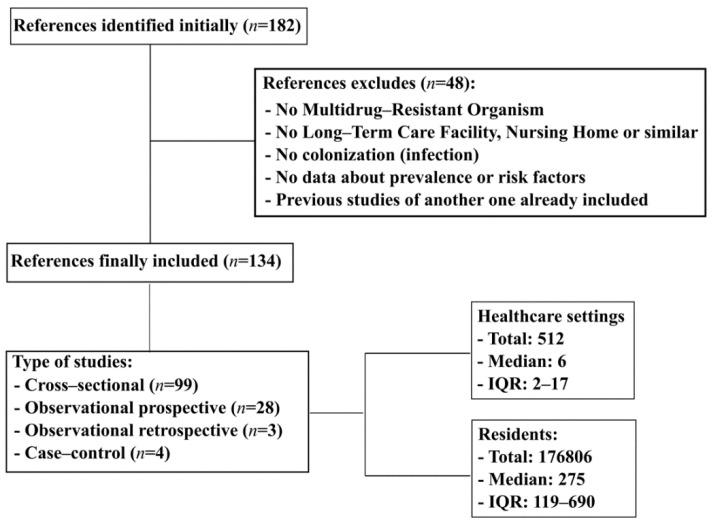
Flowchart search strategy. IQR, interquartile range.

Initially, 182 references were selected. Among these references, 48 were excluded as follows: (i) studies that did not include MDROs, NH, or LTCF (*n* = 18); (ii) studies reporting infections by MDROs instead of colonization (*n* = 17); (iii) limited information about the prevalence of or risk factors for colonization by MDROs (*n* = 9); and (iv) study population duplicated in included studies (*n* = 4). Ultimately, 134 references were included (Figure 1). Studies included in this review were published from 1987 to 2020). The most frequent MDRO analyzed was MRSA (*n* = 88, 65.6%) [1,12,13,14,15,16,17,18,19,20,21,22,23,24,25,26,27,28,29,30,31,32,33,34,35,36,37,38,39,40,41,42,43,44,45,46,47,48,49,50,51,52,53,54,55,56,57,58,59,60,61,62,63,64,65,66,67,68,69,70,71,72,73,74,75,76,77,78,79,80,81,82,83,84,85,86,87,88,89,90,91,92,93,94,95,96,97,98] followed by ESBL *Enterobacterales* (*n* = 51, 38.0%) [1,14,16,18,19,20,21,22,23,25,31,33,34,39,57,62,73,75,76,82,83,85,89,97,99,100,101,102,103,104,105,106,107,108,109,110,111,112,113,114,115,116,117,118,119,120,121,122,123,124,125] (*E. coli* and *K. pneumoniae*), VRE (*n* = 36, 26.8%) [1,13,14,18,20,21,22,23,25,28,31,33,34,37,38,39,57,58,59,60,64,65,66,73,74,76,82,83,89,97,106,109,126,127,128,129], CR *Enterobacterales* (*n* = 27, 20.1%) [1,13,18,19,20,21,22,34,60,67,73,82,83,97,100,103,104,108,109,110,113,118,130,131,132,133,134], *C. difficile* (*n* = 11, 8.2%) [19,37,106,109,131,135,136,137,138,139,140], MDR *A. baumannii* (*n* = 11, 8.2%) [13,20,28,37,60,64,67,74,82,85,141], and MDR *P. aeruginosa* (*n* = 8, 5.9%) [13,20,34,37,64,67,85,142]. The types of studies were as follows: 99 (73.8%) cross-sectional studies [1,15,16,17,19,26,28,29,30,31,32,34,35,37,39,40,42,43,44,46,47,50,52,54,55,57,59,60,61,62,65,68,69,70,71,72,73,75,77,78,79,80,81,82,83,84,85,87,88,89,91,92,93,96,97,98,99,100,101,102,103,104,105,106,107,108,109,111,113,116,117,118,119,120,121,122,123,124,125,126,128,129,131,132,133,134,135,137,138,140,141,143], 28 (20.9%) observational prospective studies [12,13,14,18,27,33,36,38,41,45,48,51,53,56,58,66,67,76,90,94,95,110,112,115,127,136,139,144], 3 (2.2%) observational retrospective studies [63,64,114], and 4 (2.9%) case-control studies [74,86,130,142]. The MDRO samples were isolated from various sources: nasal (*n* = 79, 58.9%) [12,14,15,16,18,34,36,38,40,41,42,43,45,46,47,48,49,50,51,52,53,54,55,56,57,58,59,60,61,62,63,64,65,66,67,68,69,70,71,72,73,74,75,77,78,79,80,81,82,83,84,85,86,87,88,90,91,92,93,94,95,96,97,99], perianal (*n* = 79, 58.9%) [12,14,17,18,19,20,21,22,23,25,28,29,31,32,33,34,38,41,46,47,51,56,57,58,59,60,64,65,66,67,73,74,75,76,78,82,83,85,93,94,99,100,102,103,104,105,106,108,109,110,111,112,113,115,116,117,118,119,120,121,122,124,126,127,128,129,130,131,132,133,134,135,136,137,138,139,140,142,144], skin (*n* = 33, 24.6%) [12,20,21,22,23,24,28,29,32,33,39,41,43,50,54,60,64,65,66,69,71,72,73,74,79,86,87,92,97,99,105,127,139], oropharynx (*n* = 24, 17.9%) [15,20,21,24,25,29,30,31,32,33,41,47,57,62,64,65,66,74,83,84,85,93,113,125], ulcers (*n* = 28, 20.9%) [12,14,23,29,30,31,32,33,36,41,43,47,51,56,57,63,64,65,66,71,74,78,80,81,83,93,102], urine (*n* = 15, 11.2%) [12,14,16,20,21,22,32,43,80,101,102,104,114,123,124], devices (*n* = 14, 10.4%) [12,18,23,29,30,33,41,64,65,66,68,80,93,102], and sputum (*n* = 3, 2.2%) [43,78,141].

The studies were conducted in several continents, mainly Europe (*n* = 70, 52.2%) [1,13,15,16,17,18,19,20,21,22,25,30,31,32,34,36,37,39,40,46,47,48,49,50,52,53,55,57,62,63,68,70,71,72,75,76,77,79,80,83,84,85,86,88,91,92,93,98,99,101,103,104,105,107,108,109,110,114,115,116,117,118,119,120,121,123,124,136,140], North America (*n* = 41, 30.6%) [12,14,23,24,27,29,33,38,41,42,43,44,45,51,58,59,64,65,66,73,74,78,89,90,94,97,102,109,122,127,129,130,131,133,134,137,138,139,141,142,143,144], and Asia (*n* = 15, 11.2%) [26,28,35,54,60,67,69,87,95,96,100,111,112,126,132]. Seven studies reported data from Oceania (5.2%) [56,81,82,106,113,125,128] and only one from South America (0.7%) [61]. None of the studies were performed in Africa.

### 2.2. MDRO Prevalence

The overall prevalence of colonization with MDROs around the world varies between the different pathogens studied. For ESBL *Enterobacterales*, the median prevalence reported was 11.6%, which increased to 15.0% in the case of ESBL *E. coli* and decreased to 2.9% for ESBL *K. pneumoniae*. The overall prevalence of CR *Enterobacterales* was 0.8%. Among other MDR Gram-negative bacteria, such as P. aeruginosa and *A. baumannii*, the prevalences reported globally were 1.3% and 5.8%, respectively. In the case of Gram-positive bacteria, MRSA showed the highest prevalence at 13.2%, followed by *C. difficile* 5.1% and VRE 1.5% (Table 1).

**Figure 2:**
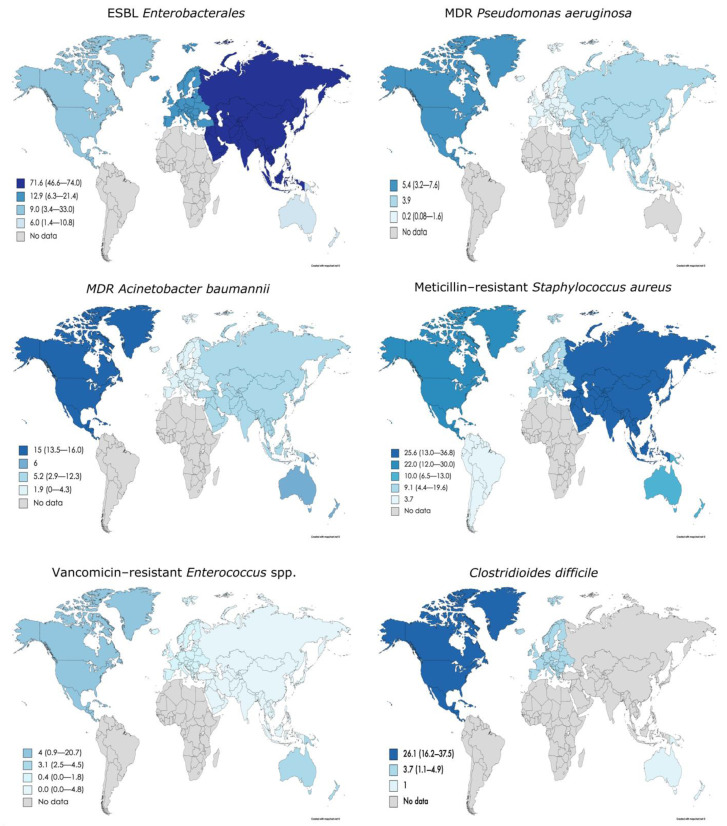
Prevalence of different MDRO in LTCF around the world. Maps were created through the online tool Mapchart.net (https://mapchart.net/world.html, accessed on 23 November 2020). ESBL, extended-spectrum ß-lactamase; MDR, multidrug-resistant.

This prevalence appears to vary between continents. Asia reported the highest prevalence of ESBL *Enterobacterales* [100,111,112], CR *Enterobacterales* [60,67,100,132] and MRSA [26,28,35,54,60,67,69,87,95,96]. In contrast, the lowest prevalence of ESBL *Enterobacterales*, CR *Enterobacterales* and MRSA have been reported in Oceania [82,106,113,125], Europe [1,13,18,19,20,21,22,34,83,103,104,108,109,110,118] and South America [61]. With respect to MDR P. aeruginosa [64,142], *A. baumannii* [64,74,141], VRE [14,23,33,38,58,59,64,65,66,73,74,89,97,127,129] and *C. difficile* [131,137,138,139], North America reported the highest prevalence. However, remarkably, Asia has the lowest prevalence of VRE [28,60,126] (Table 1, Figure 2).

### 2.3. MDRO Co-Colonization

Co-colonization with multiple MDROs seems variable among LTCF. For example, Jans et al. [31] reported a low rate of co-colonization (0.8%) with ESBL *Enterobacterales* and MRSA among 2791 residents from 60 LTCF. On the other hand, Ludden et al. [18] found that the prevalence of co-colonization with MRSA and ESBL *Enterobacterales* increased to 39%, although the authors only analyzed one LTCF with 64 residents. Heinze et al. [66] reported that 13.2% of the residents were co-colonized with both MRSA and VRE. When MDROs were considered, the prevalence of co-colonization ranged from 5.5% to 21% for at least two MDROs [57,85,142]. All of these studies suggest that LTCF may act as reservoirs for MDROs, through which transmission may occur via resident–resident interactions or resident–healthcare worker interactions.

### 2.4. Changes in MDRO Prevalence Over Time

**Figure 3:**
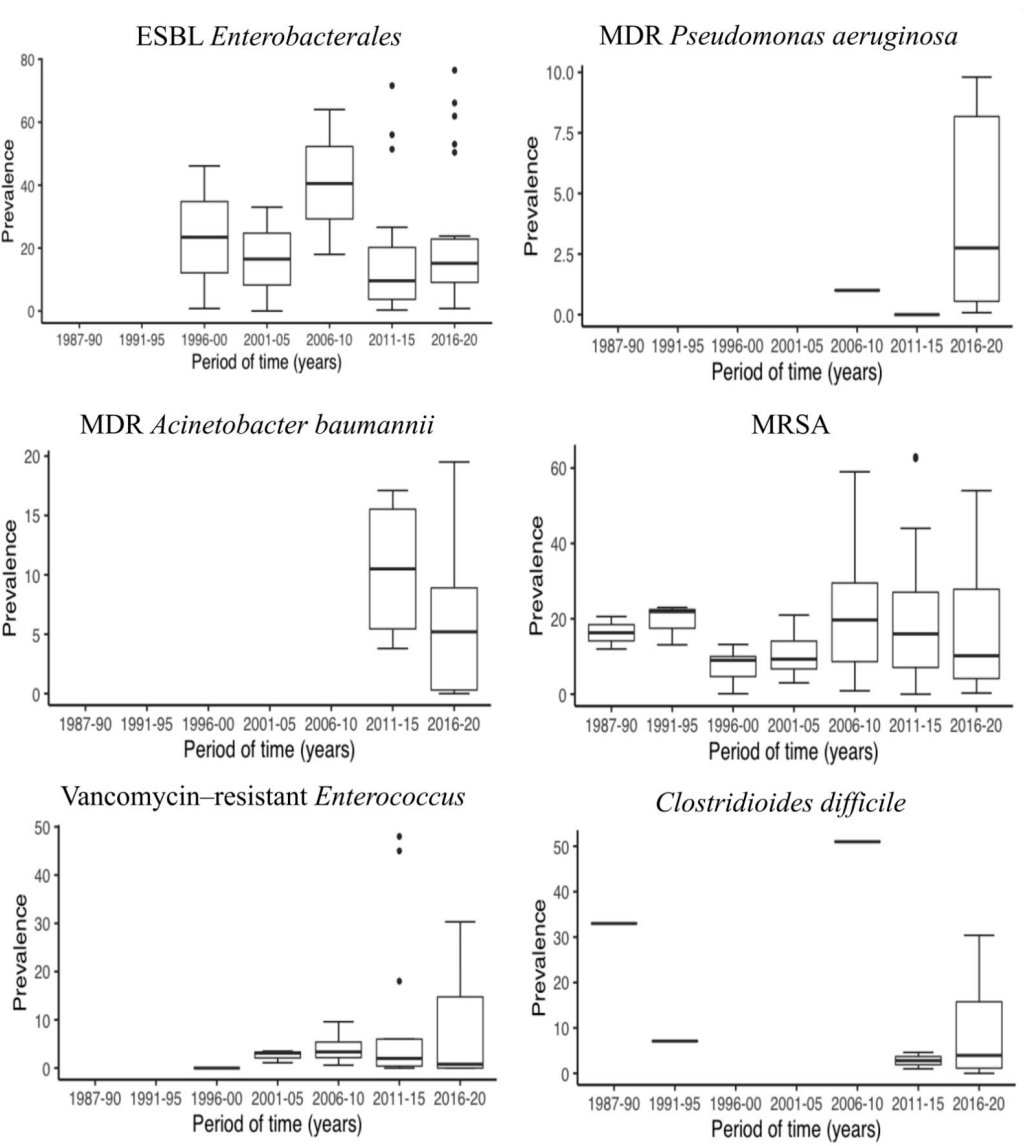
Prevalence of different MDRO in LTCF in 5-year periods. ESBL, extended-spectrum ß-lactamase; MDR, multidrug-resistant; MRSA, methicillin-resistant Staphylococcus aureus.

In the last decade, there has been a remarkable increase in the number of publications on MDR prevalence and LTCF. Figure 3 shows a representation of the median prevalence of MDROs within 5-year periods from 1985 to 2020. In order to be able to observe if there have been changes in the prevalence of MDROs over time, we established a cut-off point in 2015 and compared two time periods (until 2015, including and after 2015). Thus, we had a balanced number of publications on both groups, for each MDRO (Table 2). The ESBL *Enterobacterales* prevalence increased from 10.5% [14,18,22,23,25,31,33,39,57,75,76,82,85,89,99,101,102,104,106,107,111,112,116,119,121,122,123,124,125,143] to 15.1% [1,16,19,20,21,34,62,73,83,97,100,103,105,108,109,110,113,114,115,117,118,120], but this increase seems to be due to ESBL *K. pneumoniae* (0.7% vs. 4.2%) rather than ESBL *E. coli* (18.0% vs. 14.4%). In contrast, the prevalence of CR *Enterobacterales* decreased from 2015 onward (2.9% vs. 0.8%) [18,22,82,104,132,133] vs. [1,13,19,20,21,34,60,67,73,83,97,100,103,108,109,110,113,118,130,131,134], as did that of MDR *A. baumannii*, from 10.5% to 5.2% [74,82,85,141] vs. [13,20,28,37,60,64,67]; that of VRE, from 2.8% to 0.6% [14,18,22,23,25,31,33,38,39,57,58,59,65,74,76,82,89,106,126,127,128,129] vs. [1,13,20,21,28,34,37,60,64,66,73,83,97,109] ; that of MRSA, from 16.0% to 9.6% [14,17,18,22,23,25,26,27,29,30,31,32,33,35,36,38,39,40,41,42,43,44,45,46,47,48,50,54,56,57,58,59,63,65,68,69,71,72,75,76,77,78,79,80,81,84,85,86,89,90,91,93,94,95,96,98] vs. [1,13,15,16,19,20,21,24,28,34,37,49,55,60,61,62,64,66,67,70,73,83,87,88,92,97]; and that of *C. difficile*, from 7.1% to 3.9% [106,137,138,139,140] vs. [19,37,109,131,135,136]. Conversely, the prevalence of MDR P. aeruginosa experienced a slight increase in recent years (0.5% vs. 2.75%) [85,142] vs. [13,20,34,37,64,67] (Table 2). At a more individual level, Kohler et al. [13] analyzed changes in the prevalence of MDROs between 2007 and 2017 in NH from Switzerland. The authors reported an increase from 1.6% to 7.8% in ESBL *K. pneumoniae* and from 2% to 8% in MDR P. aeruginosa. Furthermore, a decrease from 34.3% to 25.9% was observed in MRSA prevalence. However, in contrast with the global data from this review, ESBL *E. coli* showed an increase from 5.4% in 2007 to 21.6% in 2017. Min et al. [33], in a one-year period of study, also reported an increase in the prevalence of ESBL-*Enterobacterales* from 36% to 39% and of VRE, from 10% to 13%. However, in contrast with the previous study, they observed an increase in the prevalence of MRSA, from 23% to 43%.

### 2.5. Risk Factors for Colonization

#### 2.5.1. Reside in Nursing Homes

Residing in NH and other LTCF was reported in many studies to be a risk factor for MDRO colonization [28,37,99]. A point-prevalence study analyzing MDRO prevalence in LTCF, rehabilitation clinics, and homecare facilities found that LTCF and homecare services correlated with a much higher prevalence of MDROs and *C. difficile* than rehabilitation clinics [37]. In a prospective study performed in France to identify and compare residents of NH or LTCF colonized with bacteria (defined as exposure to the risk of carrying MDROs) with those living at home (defined as non-exposed), the rate of MDRO carriage was found to be three times higher among institutionalized patients [99].

In a cross-sectional study conducted in a 342-bed hospital and 4 LTCF in Spain, at hospital admission, the prevalence of MRSA colonization was found to be around five times more likely (odds ratio (OR) 4.84 95% CI 1.00–23.51) among patients referred from an LTCF or residential center than among patients from the community [72]. In Singapore, the admission prevalence of MRSA colonization was 41% (78/190) for those from NH and 6.0% (885/14,849) among non-nursing home residents (relative risk (RR) 6.89 95% CI 5.74–8.26). In this study, the fraction of the MRSA burden upon admission attributable to NH residency was 6.92% [35]. In a German study, the prevalence of intestinal colonization by *C. difficile* among 240 NH residents was higher than that among 249 volunteers living outside the LTCF (4.6% vs. 0.8%, *p* = 0.02) [140].

Thus, residing in LTCF seems by itself to be a risk factor for colonization by MDROs. However, differences in NH infrastructure need to be considered. Specifically, the presence of communal areas and multi-bed resident rooms could impact the transmission risk inside such areas [109].

#### 2.5.2. Age

Older age was also established in many studies as a risk factor for MRSA colonization based on bivariate analysis [13,20,26,61,79,98]. In most studies, age was analyzed as a categorical variable. An analysis of national surveillance data over an 11-year period between 2007 and 2017 in Swiss NH, including a total of 9940 residents, established three categories (<70, 70–85, and >85) that increased the colonization risk from age 70 onward [13]. Similar results were obtained in Ireland for residents >80 years [98] and in four Italian LTCF fixing the cut-off at >86 years [20]. However, in these studies, the rates reported as an increased risk of MDRO colonization were two-fold lower and lacked statistical significance in the multivariate analysis.

Some studies reported age as an independent risk factor via multivariate analysis [22,26,80]. In Northern Ireland, a point-prevalence study of 45 NH including 1111 residents, based on a reference age of <60 years, found that those aged ≥90 were around nine times more likely to be colonized in the adjusted analysis (OR 8.77 95% CI 2.86–26.86; *p* < 0.001) [80]. An Italian study reported OR 4.63 (95% CI 1.12–19.1; *p* = 0.034) in residents with age ≥86 years to be colonized by ESBL producers [22], and in Taiwan, MRSA colonization was more frequent in residents aged ≥60 years (OR 2.164 95% CI1.168–4.001; *p* = 0.013) [26].

No difference was established by age as a continuous variable in a study including 14 NH in Taiwan; however, an age >60 years was significantly associated with MRSA colonization (OR 2.164 95% CI 1.168–4.011) [26]. An age ≥86 years was related to ESBL colonization, but not to MRSA colonization in a point-prevalence study of MRSA, VRE, ESBL *Enterobacterales*, CR, and high-level AmpC activity in a 120-bed LTCF attached to the regional hospital in Bolzano, Italy [22].

Therefore, older patients were generally around twice as likely to be colonized than younger patients across the studies; however, some studies found no differences up to 90 years of age based on multivariate analysis. In addition, the different categories used in the studies prevent us from establishing a cut-off or fixed age that is more likely to be colonized by MDROs (Table 3).

#### 2.5.3. Sex

The risk of MDRO colonization was higher in males than in females across the studies [13,20,31,43,47,49,77,98] (Table 3). An Italian point-prevalence study of 340 residents in four LTCF found male sex to be an independent risk factor for MRSA colonization with an OR of 2.31 (95% CI 1.16–4.59); however, male sex was not found to be an independent risk factor for ESBL colonization in this study [20]. In Ireland, a study including 6 NH and 786 residents obtained similar results for MRSA colonization, with an OR 2.18 95% CI (1.28–3.73) [98]. Other studies reported male sex to be around 1.5 times more likely than the female sex to be colonized by MRSA [77,80].

In Belgium, a multilevel logistic regression analysis of individual risk factors for ESBL *Enterobacterales* carriage among 2457 residents of 60 NH determined an OR of 1.5 (95% CI 1.1–2.1) associated with the male sex [31]. Other authors found similar results for ESBL colonization in Switzerland [13].

The reason why male sex is a risk factor remains unknown. However, the higher frequencies of other risk factors among male residents, who have more co-morbidities compared to female residents, likely predisposed men to have around 1.5 times greater risk than women for MDRO acquisition in most studies.

#### 2.5.4. Underlying Diseases: Dementia (Lower Cognitive Status), Diabetes, Cancer and Chronic Wound (Pressure/Decubitus Ulcer)

Analyzing the impact of underlying diseases on MDRO colonization is not a straightforward task, since miscellaneous baseline diseases are included in many studies, which makes it challenging to draw conclusions. In some studies, underlying diseases were ranked using the Charlson index or co-morbidity score [74,79]. Residents with a Charlson index of ≥2 were 1.5 times more probable to be colonized by MRSA in a study performed on two communities in Spain, including nine LTCF with a total of 1586 beds [79].

In the same way, some studies analyzed diseases such as dementia as encased variables within chronic cerebral conditions to rank their Global Deterioration Score (ranging from 0—normal to 7—end-stage dementia) [28,58,96]. Two studies in Hong Kong found dementia, encased with other chronic cerebral conditions such as cerebrovascular accidents and Parkinson’s disease, to be independent risk factors for MRSA colonization based on multivariate analysis [28,96]. Other studies included the level of dementia, categorized as dementia or advanced dementia, or referred to low cognitive status. A cross-sectional study performed in four nursing units in a 648-bed LTCF in Boston (USA) identified advanced dementia, but not dementia as a risk factor for MDR Gram-negative bacteria colonization [59].

In addition to dementia, cancer and diabetes were evaluated as possible risk factors for MDRO colonization in many studies [17,20,66,74,112]. Diabetes was found to be an independent risk factor for MDR *A. baumannii* colonization in residents with an indwelling device (urinary catheter and/or feeding tube) in a nested case-control study within a multicenter prospective intervention trial in the USA [74]. Other authors found diabetes to be an independent predictor of co-colonization by MRSA and VRE [66], as well as ESBL colonization [112]. However, there is no strong evidence for the association between diabetes and MDRO colonization, since other studies found no supporting information for diabetes as an independent risk factor for MDRO colonization based on multivariate analysis [28,60]. This is also the case for cancer. A point-prevalence survey in two LTCF with 551 residents in Italy established that the risk of MRSA carriage was increased by six times in patients with cancer [17]. However, cancer was found to be an independent risk factor for ESBL colonization, but not for MRSA colonization in another Italian point-prevalence study performed in four LTCF, including 340 residents [20]. In Hong Kong, a study including 1408 residents from 28 NH determined an increased risk among cancer patients for CR *A. baumannii* colonization, but not for MRSA colonization in a bivariate analysis [28]. Dementia, diabetes mellitus, and cancer are the most frequent underlying diseases analyzed in relation to the risk of MDRO colonization across the various studies. However, chronic wounds (or pressure/decubitus ulcers) showed the clearest relationship to MDRO colonization [57,82]. The presence of chronic wounds is the principal baseline condition evaluated in MRSA colonization studies [28,31,43,51,79,99], and strong evidence has shown chronic wounds to be an independent risk factor for MRSA colonization, increasing the risk by around two times in studies performed in Spain, Belgium, and the USA [31,43,79]. In addition to MRSA colonization, studies have reported decubitus ulcers to be an independent risk factor for VRE colonization [97,127], as well as MRSA and VRE co-colonization [66].

In short, the presence of wounds is one of the principal underlying conditions related to MDRO colonization across the studies, mainly in MRSA studies. On the other hand, the information is controversial for diabetes and cancer. There is stronger evidence supporting dementia. However, like age, no cut-off dementia level has been established for colonization by MDROs. Generally, when the severity of illness or fragility increased the odds of MDROs, colonization also increased [43,66] (Table 3).

#### 2.5.5. Dependence or Disability

Lower functional status has been independently associated with MDRO colonization in many studies [18,19,20,22,28,30,31,57,62,65,74,83,97,112] (Table 3). However, similar to age and dementia, establishing a level of dependence for the probability of MDRO colonization is difficult—in this case, due to the variability of the scores used across the studies. Some studies described functional status according to the Katz Index of Independence in Activities of Daily Living (ADL) and the Physical Self-Maintenance Score (PSMS), with a score of 6 indicating total dependence in six major daily activities (i.e., bathing, dressing, using the toilet, transferring, continence, and feeding) [58,59,82]. A nested case control study within a multicenter prospective intervention trial including 168 residents (25 cases vs. 143 controls) in the USA found that residents with a PSMS score >24 were associated with a 5.1 times greater likelihood (95% CI 1.8–14.9) of MDR *A. baumannii* colonization [74].

Another score frequently used is the Barthel index, which ranges from total dependence (<20 points) to independence (100 points), usually represented as percentage [22,72,79,85]. In an Italian study, the most significant risk factor for MRSA and ESBL producers and all resistant bacteria among LTCF residents was chronic immobility, based on a Barthel index score of 0 [22]. Another mobility index is the Karnofsky index, which is often used to evaluate the functional status in LTCF [99,127] and is useful as a mortality predictor. A Karnofsky index of ≤50% indicates a higher mortality risk in the following six months and means that the resident is bedridden for at least 50% of the day. A Karnofsky index of ≤50% was the only risk factor that enhanced the institutionalized carrier rate in a prospective study conducted in France to identify and compare the residents of NH and LTCF (defined as exposure to the risk of carrying MDROs) with those living at home (defined as non-exposed) [99]. Some studies used a French index known as GIR to evaluate the level of dependency (varying from 1 to 6, with an index close to 1 indicating a high level of dependency) [46,118]. A GIR score of <3, corresponding to severe disabilities, was associated with MRSA carriage in a prevalence study performed in a 120-bed LTCF containing three units and belonging to a French teaching hospital [46].

Apart from the index, dependence categories among studies include autonomous mobility vs. non-autonomous vs. bedridden [19]; ambulant (with or without help) vs. bedridden [31,83,105]; and ranking the dependence level as low, medium, or high [91].

Despite the lack of a dependence level cut-off related to MDRO colonization, there is seemingly compelling evidence to indicate than an increase in the level of dependence is associated with an increase in the risk to be colonized by MDROs.

#### 2.5.6. Medical Devices: Indwelling or Invasive Devices, Urinary Catheters, and Gastrointestinal Tubes (Feeding or Percutaneous Enteral Gastrostomy Tubes)

Medical devices, feeding/percutaneous enteral gastrostomy (PEG) tubes, and urinary catheters are considered primary potential risk factors across the studies for all MDROs [82] (Table 3). In an Australian point-prevalence study of four co-located LTCF, including 115 residents, the presence of medical devices was observed to be an independent risk factor for MDRO colonization, with an OR of 5.58 (95% CI 1.34–23.32) [82]. Specifically, for MRSA colonization, indwelling or invasive devices increased the risk by around three times among the 443 residents in the seven NH in China [87]. Similar results were obtained in a Spanish study, including nine LTCF [79] and in a cross-sectional prevalence survey of 708 residents from 39 care homes in the United Kingdom [77]. Patients with indwelling devices in place were 5.5 times as likely as those without devices to suffer MRSA and VRE co-colonization (95% CI: 2.2–13.7) [66].

In some studies, differences were noted in the types of medical devices (nasogastric tubes, PEG tubes, tracheostomy tubes, and urinary catheters) in relation to ESBL or MRSA colonization based on bivariate analysis [20,22,97]. However, these differences were neither uniform across the studies, nor in the multivariate analyses. The presence of urinary catheters was found to be an independent risk factor for MRSA and VRE, but not ESBL colonization in a serial point-prevalence study (based on six point-prevalence samples of 50 residents, each for at least two weeks) at three NH in southern California (USA) [97]. However, other studies reported urinary catheters to be an independent risk factor for ESBL but not for MRSA colonization [20,57]. Gastrointestinal devices have been associated with an increased risk of MRSA, ESBL, and CR *Enterobacterales*, as well as CR *A. baumannii* colonization, in different studies [28,73,102,109]. Other devices like ventilators were found to be independent risk factors for CR *A. baumannii* and CR *Enterobacterales* colonization [132,141].

The presence of medical devices, regardless of the type of devices or MDROs, was found to be a common risk factor for MDRO colonization across the studies. Mody et al. designed a multimodal-targeted infection program to reduce the prevalence of MDRO device-related infections. The Targeted Infection Program included the following: (1) preemptive barrier precautions; (2) active surveillance for MDROs and infections with data feedback; and (3) NH staff education on key infection prevention practices, as well as the promotion of good hand hygiene. The results of this randomized clinical trial showed a 23% reduction in MDRO prevalence, including MRSA, VRE, and Gram-negative bacteria resistant to either ciprofloxacin or ceftazidime in the intervention group [145].

#### 2.5.7. Antibiotics Use in the Preceding Months

In a large observational study conducted in north-eastern France, resistance to amoxicillin/clavulanate, fluoroquinolones, and ceftriaxone from *Enterobacterales* (*E. coli*, Proteus mirabilis, and *K. pneumoniae*) cultured from urine samples collected in 2014–17 was around 40% higher among the NH residents than among older adults living in the community [114].

Antibiotic consumption is the main modifiable factor necessary to avoid an exponential increase in antimicrobial resistance. Wang et al. described the influence network comprising antibiotic treatment, MDRO colonization, and infection by leveraging active surveillance and antibiotic treatment data for 234 NH residents and demonstrated the importance of designing strategies that account for a complex set of inter-dependencies among different MDROs and the antibiotics that influence their spread [64]. Thus, ecological antibiotic prescriptions must come first. Fluoroquinolone use has been reported to be an independent risk factor for MDRO colonization in multiple studies [20,30,38,83,84,116]. Importantly, ASPs would help encourage the correct use of antibiotics. In the Netherlands, a study in 64 LTCF with at least 50 beds (excluding rehabilitation) determined higher consumption (each extra 50 DDD/1000 residents/day nearly doubled carriage) as the strongest predictor of ESBL *E. coli* carriage based on multilevel multivariable logistic regression (OR 1.8, 95% CI 1.1–3.0) [16]. In Northern Ireland, residents who were found to carry MDR *E. coli* were more likely to have been prescribed fluoroquinolones or trimethoprim. In this study, the number of days of fluoroquinolone use was found to be an independent risk factor for MDR *E. coli* colonization (OR 1.33, 95% CI 1.04–1.69) [116].

Previous antibiotic use has also been reported to be a risk factor for MDRO colonization [22,31,35,79,126] (Table 3). Specifically, exposure to amoxicillin/clavulanate or fluoroquinolones was the most frequent factor analyzed, since these are broad-spectrum antibiotics [1,30]. The use of ß-lactam/ß-lactamase inhibitors in the preceding six months was also found to be an independent risk factor for MRSA colonization (OR 2.34 95% CI 1.44–3.82) in a study of 2322 subjects from 28 NH in the Hong Kong west district [28]. Documented or probable antibiotic use in the 60 days before admission was correlated with VRE colonization (OR 4.2 95% CI 1.4–12.5; *p* = 0.008) in hospitalized residents of the Rush-Presbyterian–St. Luke’s Medical Center in Chicago (USA) [127].

*C. difficile* is not defined as an MDRO; however, *C. difficile* was included in this review due to its impact on healthcare management in LTCF, specifically as a result of inappropriate antibiotic use. A meta-analysis on the prevalence of and risk factors for asymptomatic carriers of toxigenic *C. difficile* in LTCF determined that antimicrobial use in the previous three months was associated with around a four-times greater risk of being colonized [146].

#### 2.5.8. Hospital Admission in the Previous 12 Months, Any Department

In studies performed in Belgian and American NH, hospital admission in the previous 12 months increased the risk of MRSA colonization by up to four times [36,83]. A Belgian study including 2953 residents of 60 highly skilled NH observed around a two-times greater risk of MRSA and ESBL colonization among residents who were hospitalized in the previous 12 months [30]. For MRSA colonization, this risk increased by up to nine times in a Brazilian study analyzing previous hospitalization over the preceding six months [61] and by around three times for VRE colonization in a cross-sectional survey among 1215 residents of LTCF in Israel [126]. Hospital admission in the previous three months was also reported to be a risk factor for MDRO colonization [85].

There are no data on how long after hospital admission this risk increases. Thus, it remains unknown if hospital admission in the previous three months could entail greater risk for colonization than hospitalization in the last year. In the United Kingdom, a hospitalization duration of more than 10 days during the previous two years was independently associated with MRSA colonization; however, in this study, less than 10 days of hospitalization was not associated with MRSA colonization [77]. In a 351-bed community LTCF for older adults in Slovenia, multiple hospital admissions, defined as more than one instance of hospitalization in the three months before the investigation, increased the risk of MRSA colonization by around six times. In this study, a stratified analysis found that hospitalized residents with diabetes were 14 times more likely to be colonized compared to non-hospitalized diabetic residents [86].

In conclusion, previous hospitalization could be considered a risk factor for MDRO colonization among LTCF residents. However, to what extent this risk could be increased by days of hospitalization remains unknown.

#### 2.5.9. Previous Colonization by MDRO

There is controversy about whether previous colonization with MDROs could be a risk factor for being colonized with an MDRO (Table 3). Two studies performed in Amsterdam [34] and Spain [85] showed that previous colonization with any MDRO was an independent risk factor for colonization with other MDROs (OR 4.7 (1.8–11.7) and (OR 10.89 (3.65–32.43), respectively). Another study performed by McKinnell et al. [97] showed that previous MDRO history was independently associated with colonization by MRSA [OR 3.6 (1.5–8.3)] and ESBL *Enterobacterales* [OR 4.1 (1.6–10.4)]. In contrast, Terveer et al. [109] did not find any association between previous colonization by MDROs and colonization by ESBL *E. coli*. At the individual pathogen level, previous colonization with MRSA has been reported as an independent risk factor for colonization with MRSA [42,43,57,83,84]. In the case of ESBL *Enterobacterales*, several studies reported an association between previous colonization with ESBL *Enterobacterales* and colonization with ESBL *Enterobacterales* [31,73,97]. however, one study performed by Latour et al. with 1447 residents from 29 LTCF did not find such a relationship [83]. The persistence of colonization was measured by Ludden et al. [18] in a study with 64 residents from one LTCF. Out of the 24 residents with ESBL *E. coli*, 75% remained positive for six months, and 42% remained positive for one year. Regarding VRE colonization, data from the SHIELD Orange Country Project performed by McKinell et al. [73] found that previous VRE history was associated with colonization by ESBL [OR 2.6 (1.3–5.3)] and CR *Enterobacterales* [OR 4.8 (1.3 17.9)]. Furthermore, Heinze et al. [66] reported that being previously colonized with MRSA or VRE was a significant predictor for becoming co-colonized by MRSA/VRE [hazard ratio (HR) 17.0 (5.3–54.6)] and [HR 6.3 (2.2–18.2)].

Therefore, previous colonization with MRSA is clearly a risk factor for colonization with MRSA. In other MDROs, this relationship is not completely clear and could depend on other factors, in addition to previous colonization, that differ between studies. There is evidence that asymptomatic carriers may contribute to transmission and that implementing control measures is necessary to avoid an increase in reproductive numbers [146,147,148].

## 3. Materials and Methods

### 3.1. Search Strategy

A search was performed on the search engine PubMed and database Scopus for studies describing the prevalence of MDROs in LTCF or NH. The search terms used were related to microorganisms, such as “multidrug-resistant organism”, “extended-spectrum ß-lactamase *Enterobacterales*/*Enterobacteriaceae*”, “extended-spectrum ß-lactamase *Escherichia coli*”, “extended-spectrum ß-lactamase *Klebsiella pneumoniae*”, “carbapenem-resistant *Enterobacterales*/*Enterobacteriaceae*”, “multidrug-resistant *Pseudomonas aeruginosa*”, “multidrug-resistant *Acinetobacter baumannii*”, “methicillin-resistant *Staphylococcus aureus*”, “vancomycin-resistant *Enterococcus*”, and “*Clostridium/Clostridioides difficile*” and those related to the site, such as “long term care facility” and “nursing home”. The reference lists of the included studies were searched to identify further studies.

### 3.2. Study Selection and Eligibility Criteria

Inclusion criteria were defined as studies that analyzed and reported data on the prevalence and/or risk factors for the acquisition of MDROs in LTCF or NH. All types of studies were included, except for reviews. Restrictions on the year of publication were not established. Exclusion criteria included when studies reported data on non-MDROs and/or when the site was different from LTCF or NH healthcare settings (e.g., veterans’ affairs centers, which provide acute inpatient and outpatient care) and/or when the studies analyzed infection by MDROs instead of colonization. Notably, the studies were selected and screened by two independent reviewers (ARV and CMG). Disagreements were resolved by consensus through discussions.

### 3.3. Definitions

The microorganisms selected for inclusion in this review are those commonly associated with MDR, such as *Enterobacterales*, especially *E. coli* and *K. pneumoniae, P. aeruginosa*, *A. baumannii*, *S. aureus*, and *Enterococcus* spp. We also included data on *C. difficile* colonization, because *C. difficile* is a healthcare-associated microorganism.

Focusing on antimicrobial resistance, ESBL was defined differently between the studies analyzed depending on the methodology and interpretation criteria used. To avoid confusion and the use of several terms, we grouped studies that reported both ESBL and resistance to third or fourth generation cephalosporins under the term “ESBL”. For *P. aeruginosa* or *A. baumannii*, MDR was considered as resistance to carbapenems and/or resistance to three or more classes of antibiotics comprising cephalosporins, carbapenems, quinolones, and aminoglycosides.

For the healthcare setting, LTCF and NH are similar terms used to describe sites that provide living accommodations for people who require the on-site delivery of supervised care 24 h per day, 7 days per week. Importantly, not all residents of LTCF are older adults [149].

## 4. Conclusions

The prevalence of MDROs in LTCF varies across the world, showing great differences across continents. Asia has the highest prevalence of ESBL *Enterobacterales*, CR *Enterobacterales*, and MRSA, while North America has the highest prevalence of MDR *P. aeruginosa* and MDR *A. baumannii*. North America also has the highest prevalence of *C. difficile*, but this prevalence has changed over the years. In general, since 2015, the prevalence of CR *Enterobacterales*, MDR *A. baumannii*, MRSA, VRE, and *C. difficile* has experienced a decrease. In contrast, the prevalence of ESBL *Enterobacterales* and MDR *P. aeruginosa* has increased in recent years.

Living in NH and other LTCF is itself a risk factor for being colonized by MDROs. This is a non-modifiable factor and must be taken into account at hospital admission. Most risk factors for colonization with MDROs in LTCF cannot be modified. Risk factors related to age, sex, dependence, or underlying diseases should lead healthcare professionals to intensify aseptic and antiseptic measures to optimize healthcare management. However, there are modifiable factors that we can work on by designing optimal strategies to reduce MDRO propagation and improve the quality of life among the residents in LTCF. The optimal management of medical devices and healthcare for chronic wounds have a significant impact on reducing MDRO colonization. However, the principal modifiable factor is to ensure correct antibiotic use, for which the implementation of ASP would be a useful tool, following the recommendations of the CDC and other scientific and governmental organizations.

Further research is required on the epidemiology of MDRO colonization in LTCF to analyze the risk factors. Standard criteria are necessary to determine the level of dementia or dependence and thereby allow us to grade the residents as high or low risk for colonization by MDROs based on a standard score, including main aspects, such as dependence, dementia, devices, and diseases.

## Figures and Tables

**Table 1:** Prevalence of multidrug-resistant organisms in long term care facilities.

Multidrug-Resistant Organism	No. of Articles *n* = 134	Percentage of Articles *n* = 134 (%)	Microorganism Global Prevalence *n* = 134 (Median, IQR)	Prevalence in Europe *n* = 70 (Median %, IQR)	Prevalence in North America *n* = 41 (Median %, IQR)	Prevalence in South America *n* = 1 (Median %, IQR)	Prevalence in Asia *n* = 15 (median %, IQR)	Prevalence in Oceania *n* = 7 (Median %, IQR)	Prevalence in Africa *n* = 0 (Median %, IQR)
ESBL *Enterobacterales*	51	38.0	11.6 (5.5–24.5)	12.9 (6.3–21.4)	9 (3.4–33)	-	71.6 (46.6–74)	6.0 (1.4–10.8)	-
ESBL *Escherichia coli*	33	24.6	15.0 (7.7–41.4)	15.3 (7.8–41.2)	15 (2.9–30.3)	-	82.7 (50.4–86.1)	10.4 (5.6–11.2)	-
ESBL *Klebsiella pneumoniae*	22	16.4	2.9 (0.4–7.1)	4.2 (0.6–6.5)	0.2 (0.0–4.8)	-	9.1 (8.8–9.4)	1.7	-
Carbapenem resistant *Enterobacterales*	27	20.1	0.8 (0.0–4.2)	0.2 (0.0–0.9)	5.0 (2.0–7.9)	-	6.9 (1.4–14.6)	0.4 (0.3–0.5)	-
MDR *Pseudomonas aeruginosa*	8	5.9	1.3 (0.2–5.3)	0.2 (0.08–1.6)	5.4 (3.2–7.6)	-	3.9	-	-
MDR *Acinetobacter baumannii*	11	8.2	5.8 (2.2–13.5)	1.9 (0–4.3)	15.0 (13.5–16.0)	-	5.2 (2.9–12.3)	6.0	-
Meticillin-resistant *Staphylococcus aureus*	88	65.6	13.2 (6.6–25)	9.1 (4.4–19.6)	22.0 (12.0–30.0)	3.7	25.6 (13–36.8)	10 (6.5–13)	-
Vancomycin-resistant *Enterococcus* spp.	36	26.8	1.5 (0.06–6.9)	0.4 (0.0–1.8)	4.0 (0.9–20.7)	-	0.0 (0.0–4.8)	3.1 (2.5–4.5)	-
*Clostridioides difficile*	11	8.2	5.1 (1.9–24.8)	3.7 (1.1–4.9)	26.1 (16.2–37.5)	-	-	1.0	-

ESBL, extended-spectrum ß-lactamase; MDR, multidrug resistant; IQR, interquartile range.

**Table 2:** Changes in the prevalence of multidrug-resistant organisms, before and after 2015.

Multidrug-Resistant Organism	No. of Articles (2015 or Before) *n* = 90	No. of Articles (After 2015) *n* = 44	Prevalence of MDR (2015 or Before) Median (IQR)	Prevalence of MDR (After 2015) Median (IQR)	Difference (%)
ESBL *Enterobacterales*	30	22	10.5 (3.5–31.4)	15.1 (9.1–19.9)	4.6
ESBL *Escherichia coli*	19	14	18.0 (5.5–40.9)	14.4 (8.1–41.4)	−3.6
ESBL *Klebsiella pneumoniae*	10	12	0.7 (0.2–9.2)	4.2 (0.7–6.3)	3.5
Carbapenem resistant *Enterobacterales*	6	21	2.9 (0.1–7.5)	0.8 (0–1.9)	−2.1
MDR *Pseudomonas aeruginosa*	2	6	0.5 (0.25–0.75)	2.75 (0.5–8.2)	2.25
MDR *Acinetobacter baumannii*	4	7	10.5 (5.4–15.5)	5.2 (0.3–8.9)	−5.3
Meticillin-resistant *Staphylococcus aureus*	61	26	16.0 (7.8–23.3)	9.6 (3.9–25.5)	−6.4
Vancomycin-resistant *Enterococcus* spp.	22	14	2.8 (0.5–5.5)	0.6 (0.001–13.0)	−2.2
*Clostridioides difficile*	5	6	7.1 (4.6–33.0)	3.9 (1.1–15.7)	−3.2

IQR, interquartile range; ESBL, extended-spectrum ß-lactamase; MDR, multidrug resistant.

**Table 3:** Risk factors for multidrug-resistant organism colonization in long-term care facilities in the studies. Common characteristics and limitations.

Risk Factors for MDRO Colonization	Limitations and Common Characteristics
Age	An increase entails higher risk. There is not a cut-off established for colonization by MDROs.
Male sex	Confirmed in many studies by multivariate analysis
Dementia	An increase entails higher risk. There is not a cut-off established for colonization by MDROs.
Diabetes	Controversial results. Differences by MDRO type.
Cancer	Controversial results. Differences by MDRO type.
Chronic wound	Confirmed in many studies by multivariate analysis
Dependence	An increase entails higher risk. There is not a cut-off established for colonization by MDROs.
Medical devices	Confirmed in many studies by multivariate analysis
Previous antibiotic use	Confirmed in many studies by multivariate analysis
Previous hospitalization	Whether the risk could be increased by days of hospitalization is unknown.
Previous MDRO colonization	Controversial results. Differences by MDRO type.

MDRO: multidrug-resistant organism.
